# Mesenchymal Stem Cells Derived from Healthy and Diseased Human Gingiva Support Osteogenesis on Electrospun Polycaprolactone Scaffolds

**DOI:** 10.3390/bioengineering5010008

**Published:** 2018-01-23

**Authors:** Catherine Jauregui, Suyog Yoganarasimha, Parthasarathy Madurantakam

**Affiliations:** 1 Philips Institute, School of Dentistry, Virginia Commonwealth University, Richmond, VA 23298, USA; cjauregui@augusta.edu; 2 Department of Biomedical Engineering, School of Engineering, Virginia Commonwealth University, Richmond, VA 23284, USA; suyog.yoganarasimha@gmail.com; 3 Department of General Practice, School of Dentistry, Virginia Commonwealth University, Richmond, VA 23298, USA

**Keywords:** gingiva, mesenchymal stem cells, electrospinning, scaffolds, bone tissue engineering, osteogenesis, alkaline phosphatase, alizarin red

## Abstract

Periodontitis is a chronic inflammatory disease affecting almost half of the adult US population. Gingiva is an integral part of the periodontium and has recently been identified as a source of adult gingiva-derived mesenchymal stem cells (GMSCs). Given the prevalence of periodontitis, the purpose of this study is to evaluate differences between GMSCs derived from healthy and diseased gingival tissues and explore their potential in bone engineering. Primary clonal cell lines were established from harvested healthy and diseased gingival and characterized for expression of known stem-cell markers and multi-lineage differentiation potential. Finally, they were cultured on electrospun polycaprolactone (PCL) scaffolds and evaluated for attachment, proliferation, and differentiation. Flow cytometry demonstrated cells isolated from healthy and diseased gingiva met the criteria defining mesenchymal stem cells (MSCs). However, GMSCs from diseased tissue showed decreased colony-forming unit efficiency, decreased alkaline phosphatase activity, weaker osteoblast mineralization, and greater propensity to differentiate into adipocytes than their healthy counterparts. When cultured on electrospun PCL scaffolds, GMSCs from both sources showed robust attachment and proliferation over a 7-day period; they exhibited high mineralization as well as strong expression of alkaline phosphatase. Our results show preservation of ‘stemness’ and osteogenic potential of GMSC even in the presence of disease, opening up the possibility of using routinely discarded, diseased gingival tissue as an alternate source of adult MSCs.

## 1. Introduction

Bone is a unique tissue with a remarkable potential to undergo complete regeneration following injury, even in adult life. However, when the severity of the defect surpasses a critical-size, external invention in some form of grafting is required. With more than two million bone grafting procedures annually worldwide, bone represents the second most transplanted tissue next only to blood products [1]. The most common hard tissue graft is an autograft, where bone is taken from the patient's own body and reimplanted into the defect site. Autologous bone grafts are osteoconductive (provide a scaffold where bone cells can proliferate), osteoinductive (induce proliferation of undifferentiated cells and their differentiation into osteoblasts), and osteogenic (provide a reservoir of skeletal stem and progenitor cells that can form new bone). However, autografts are limited in availability, require additional invasive surgery, and have donor site morbidity [2]. Allografts derived from human donors or cadavers eliminate donor site morbidity, are osteoconductive, and are available in large quantities. However, these are associated with increased costs, laborious processing, decreased mechanical strength, limited osteoinduction, and increased risk of infection. Xenografts are cheap and are readily available but have risks of immunogenicity and slow integration [3]. Disadvantages associated with traditional sources have driven the development of synthetic bone substitutes that includes ceramics, metals, and polymers [4]. Bone tissue engineering is an alternate strategy that integrates the current advances in material science, cell biology, and bioengineering to construct viable 3-dimensional constructs that can restore structure and function to bone lost from injury or disease. The fundamental tenet of tissue engineering is the triad of scaffolds (extracellular matrix mimics), cells (including mesenchymal stem cells), and biological signaling molecules [5]. Electrospinning is a popular scaffold fabrication strategy that generates 3-dimensional, porous, nanofibrous scaffolds with a large surface area to volume ratio in a physical dimension similar to native tissues. Furthermore, electrospinning offers wide choice in the polymer composition, fiber diameter and alignment, as well as controlling scaffold density and degradation [6,7].

Adult mesenchymal stem cells (MSCs) are promising cell types in tissue engineering because of their high proliferation potential, ability to differentiate into multiple mesodermal lineages (cartilage, fat, and bone), and their ability to be manipulated in culture [8]. MSCs have been isolated from many different adult tissues, including bone marrow, adipose tissue, and peripheral blood [9]. The differentiation capabilities of MSCs into cartilage, fat, and bone from these different sources as well as their immunosuppressive properties have been widely documented [10]. However, each source also is associated with limitations: while bone marrow derived stem cells include low yield, difficulties in cell collection (harvest), aging, and limited proliferative capacity [11], adipose-derived MSCs lose genetic stability over time and are prone to tumor formation [12]. Thus, there is a need to find alternate sources for adult MSCs.

Human gingiva is a recently identified stem cell source that has the advantages of abundant tissue availability, ease of access, and scarless regeneration following harvest. MSCs isolated from human gingiva (gingiva-derived mesenchymal stem cells, GMSCs) are an attractive option in stem cell-based therapies because of their relative abundance, ease of isolation, and rapid ex vivo expansion [13]. In addition to their differentiation and self-renewal properties, GMSCs also possess unique immunomodulatory and anti-inflammatory functions [14]. Adult oral mucosal connective tissue cells, and in particular MSCs, can be easily harvested with little morbidity and possess distinct characteristics, including neural crest origin, multipotent differentiation capacity, fetal-like phenotype that may be utilized to promote tissue regeneration, and fast, scar-free wound healing [15]. Most of the abovementioned studies have used healthy gingiva as the source for isolating the stem cells.

In spite of these advantages, human periodontium (of which gingiva is a component) is one of the most commonly diseased tissues. According to the Centers for Disease Control and Prevention (CDC), half of Americans aged 30 or older have some form of periodontitis [16], a chronic infection initiated by bacteria and modulated by host immune response. A common treatment for chronic periodontitis is surgical resection of diseased tissue and thorough debridement of infected root surface. The resected diseased gingival/periodontal tissue is routinely discarded as part of surgery and is considered a medical waste. It would be of huge clinical interest to explore the potential of GMSCs derived from diseased gingiva and compare it against that of healthy tissue in the context of bone tissue engineering. In fact, one study showed that MSCs isolated from diseased gingival tissue are functionally equivalent to MSC-like cells derived from healthy gingival tissue [13].

Polycaprolactone (PCL) is a synthetic polymer on the US Food and Drug Administration’s (FDA’s) generally regarded as safe (GRAS) list. It exhibits excellent biocompatibility, complete degradation in vivo, and has been approved for drug delivery and medical devices applications. PCL has been successfully used as micro- and nano-spheres in controlled drug delivery systems [17,18] and in tissue-engineering applications [19].

Our study expands on this idea and evaluated the survival and proliferation of GMSCs from healthy and diseased gingival tissues on electrospun polycaprolactone (e-PCL) scaffolds. We began by evaluating differences in GMSCs derived from diseased and healthy human gingiva using well-established methods. Subsequently, we cultured these different populations on previously characterized e-PCL scaffolds in osteogenic and adipogenic environments to assess their ability to support osteogenesis.

## 2. Materials and Methods

The study protocol and the consent documents were reviewed and approved by the Institutional Review Board at Virginia Commonwealth University (Approval # HM 14826). All tissue culture reagents were from Invitrogen and culture vessels were from Corning, unless otherwise noted.

### 2.1. Patient and Sample Identification

We identified three healthy adults (ages 18–24) who presented to the surgery clinic for extraction of complete bony impacted third molars with healthy overlying gingiva. Patients with soft tissue impactions and pericoronitis were excluded. Gingival tissues for harvest had no clinical signs of infection/inflammation. Diseased gingival samples were tissues that were resected from patients with chronic periodontitis undergoing flap and osseous surgery (*n* = 9; ages 32–55). Soft, friable gingival tissue directly overlying the deepest periodontal pocket was identified as the diseased sample and used for this study.

### 2.2. Sample Collection and Establishment of Primary Clonal Cell Lines

Harvested gingival tissues were collected in cold, sterile saline (4 °C) and were transported to the laboratory within 30 min. Following a brief dip in 70% ethanol, tissues were washed three times in sterile phosphate buffered saline (PBS). The tissues were then finely chopped using dissecting scissors into 1 mm × 1 mm size pieces and then treated with 1 mg/mL dispase in α minimum essential medium (αMEM) for 30 min under gentle agitation at 37 °C. After brief centrifugation, the supernatant was removed and replaced with 0.66 mg/mL collagenase for 1 h at 37 °C. After centrifugation at 800 g × 5 min, the pellet was re-suspended in fresh αMEM containing 10% fetal bovine serum (FBS, Atlanta Biologicals, Flowery Branch, GA, USA) and 1% Antibiotic-Antimycotic (GIBCO) and passed through a 70 μM cell strainer, prior to seeding in 75 cm^2^ culture flasks. Flasks were then incubated undisturbed under standard culture conditions (5% CO_2_, 100% humidity, and 37 °C) for 7–10 days until confluent. Adherent cells were then isolated by trypsinization and frozen stocks prepared in Bambanker (Wako Chemicals, Richmond, VA, USA) and stored at −80 °C.

Mesenchymal stem cells derived from healthy human gingiva (hGMSCs) are referred to as Healthy samples, #A and #C. MSCs derived from diseased gingival tissues (dGMSCs) are referred to as Diseased samples, #8 and #9.

### 2.3. Routine Cell Culture

Cells were maintained in complete culture media (CCM) containing AdvanceStem™ Cell Culture Medium (HyClone, Logan, UT, USA) which contains antibiotics under conventional conditions. Media was changed every 3 days until 70–80% confluent, at which time the cells were passaged into fresh flasks, or plates/dishes as needed for assays described below. Cells between p3 and p7 were utilized for all experiments in this study. All experiments for characterizing GMSCs were done in triplicate and repeated for reproducibility.

### 2.4. Colony Forming Unit (CFU) Assay

Cells were seeded at a density of 1.0 × 10^2^ cells in 10-cm dishes and cultured under conventional conditions (*n* = 3). Non-adherent cells were removed after 2–3 days, and cells were subsequently fed every 3 days for 14 days. Colonies were then washed twice with PBS, incubated for 30 min in 0.5% crystal violet in (100% methanol), and counted.

### 2.5. Flow Cytometric Analysis

Cells were seeded at a density of 5 × 10^5^ cells in 75 cm^2^ flasks and cultured under conventional conditions for 72 h. Subsequently, cells were harvested using 0.05% trypsin- ethylenediaminetetraacetic acid and cell pellets re-suspended in PBS prior to cytometric analysis by a Human MSC analysis kit (BD Biosciences, San Jose, CA, USA). Briefly, cells were incubated with fluorescein (FITC) mouse anti-human CD90, adenomatous polyposis coli (APC) mouse anti-human CD73, PerCP-Cy5.5 mouse anti-human CD105, and a PE-conjugated negative cocktail (anti-human CD34, CD11b, CD19, CD45, and HLA-DR) on ice for 30 min. Cells were then washed in PBS and analyzed using a BD Biosciences Aria II flow cytometer.

### 2.6. Differentiation Assays

Cells were seeded in 6-well plates at a density of 5 × 10^4^ cells per well and grown to confluence under standard culture conditions. Cells were then maintained in osteogenic medium, adipogenic medium, or control CCM (HyClone), and the media were changed every three days Following 21 days incubation, wells were washed in PBS and cells fixed in 10% neutral-buffered formalin for 1 h at room temperature. Cells were then stained with either 2% Alizarin Red (Sigma-Aldrich, St. Louis, MO, USA) or 0.5% Oil Red O (Sigma-Aldrich) for 20 min at room temperature, and subsequently washed 4 times in PBS prior to microscopy. Controls for negative differentiation (cells grown in CCM stained with either stain) and negative staining (cells grown in osteogenic medium were stained with Oil Red O and cells maintained in adipogenic medium were stained with Alizarin Red) were employed.

#### 2.6.1. Scale Used to Assess Osteogenesis

0 = no mineralization over total well surface; 1 = very few cells producing mineralized nodules visible over total well surface; 2 = very few cells producing mineralized nodules but visible in several microscope fields; 3 = very few cells producing mineralized nodules but visible in majority of microscope fields; 4 = high number of cells producing mineralized nodules; 5 = positive staining of entire well surface; virtually all cells producing mineralized nodules.

#### 2.6.2. Scale Used to Assess Adipogenesis

0 = no adipocytes over total well surface; 1 = very few adipocytes visible over total well surface; 2 = very few adipocytes but visible in several microscope fields; 3 = very few adipocytes but visible in majority of microscope fields; 4 = high number of cells are adipocytes; 5 = positive staining of entire well surface, virtually all cells are adipocytes.

### 2.7. Cell Proliferation

Cells were seeded at a density of 2.5 × 10^4^ cells/well in 24-well plates and cultured until 70% confluent. Cells were starved in serum-free medium for 24 h prior to the assay and then incubated in CCM for the required times: 72 h, 1 week, 2 weeks, or 3 weeks. At each time point, the cell proliferation assay (MTS:PMS) assay was performed according to manufacturers’ instructions (Promega, Madison, WI, USA). Briefly, wells were washed twice in PBS, 100 μL of MTS:PMS reagent was dispensed in each well and was incubated for 1 h. Samples (100 μL) were subsequently taken from each well and absorbance at 490 nm read using a multi-plate reader. Dehydrogenases in metabolically active cells convert MTS into aqueous, soluble formazan, and this can be used to estimate the number of living cells in culture. A standard curve was generated for each cell line and used to normalize the data from alkaline phosphatase assays.

### 2.8. Measurement of Alkaline Phosphatase (ALP) Activity

The treatment of cells to quantify ALP activity was similar to that previously described [20]. Briefly, cellular monolayers were lysed with 100 μL of 25 mM sodium carbonate (pH 10.3), 0.1% (*v*/*v*) Triton X-100 (Lysis buffer), and treated with 200 μL of 15 mM p-nitrophenyl phosphate (di-tris salt, Sigma, Cream Ridge, NJ, USA) in 250 mM sodium carbonate (pH 10.3) and 1.5 mM MgCl_2_ (substrate buffer). Lysates were then left under conventional cell culturing conditions for 2 h. Samples were subsequently taken from each well (100 μL), dispensed into a 96-well plate, and the absorbance at 405 nm read using a multiplate reader. A blank of lysis buffer and p-nitrophenyl phosphate was used to normalize data. A series of p-nitrophenol (25–500 μM) prepared in the substrate buffer enabled quantification of product formation.

### 2.9. Electrospinning PCL Scaffolds

PCL (MW 80,000, Sigma) was dissolved in a binary solvent system of formic acid:acetic acid (1:3) at a concentration of 100 mg/mL. An electrospinning apparatus (EC-DIG, IME Technologies, Geldrop, The Netherlands) was used at optimized process conditions to generate continuous, non-woven fibers that were collected onto 18 mm diameter circular glass coverslips attached to a cylindrical drum mandrel (100 mm diameter) rotating at 100 rpm. After electrospinning, coverslips were removed from the mandrel, dried in a fume hood overnight, and stored in an airtight desiccator until use.

### 2.10. Scanning Electron Microscopy

Electrospun scaffolds were mounted on aluminum stubs using standard double-sided tape, sputter coated with platinum, and examined at an accelerating voltage of 20 KV using JEOL JSM 5610LV (JEOL, Peabody, MA, USA) scanning electron microscope. Average fiber diameter was calculated from a total of 50 randomly selected fibers from SEM images using Image J (NIH).

### 2.11. Scaffold Disinfection and Cell Seeding

The scaffolds on coverslips were disinfected with 70% ethanol for 30 min. Following disinfection, scaffolds were washed thrice with PBS (10 min each) and incubated in cell culture media overnight prior to cell seeding. Primary cell lines (2 hGMSC and 2 dGMSC) used between p3 and p7 for all assays. New batches of electrospun PCL scaffolds were generated for each experiment (cell survival, proliferation, and differentiation) and disinfected using the above-mentioned protocol. All experiments were carried out in a 12-well plate format and 100 μL of cell suspension (20,000 cells) was placed in the center of the scaffold within a 10mm diameter sterile, glass-cloning ring (Corning). Cells were allowed to attach to the scaffold for the first 24 h, before more media was added. Media was changed every 3 days for the duration of the experiments. All experiments were performed in triplicate and repeated to ensure reproducibility of results.

### 2.12. Cell Survival Using Live/Dead Assay

Live/Dead assay (Life Technologies, Carlsbad, CA, USA) was performed to evaluate scaffold cytocompatibility and early survival of GMSCs seeded onto electrospun scaffolds. At 24 h and 7 days, constructs were washed with PBS and Live/Dead stain was added at 5× concentration directly to the cell-scaffold constructs and imaged using a Nikon fluorescence microscope (10×. All images are overlaid with green and red channels indicating live and dead cells, respectively.

### 2.13. Cell Proliferation Using MTS Assay

Cell proliferation was evaluated using MTS (Promega, Madison, WI, USA) assay. Cells from the four experimental groups were seeded onto scaffolds and a modified MTS assay was performed on days 1 and 7. Scaffolds without cells served as controls. Briefly, cell seeded scaffolds were washed with PBS at designated time points and incubated with MTS reagent for 2 h. The absorbance of the supernatant was read at 490 nm using a BioTek Synergy 2 microplate reader. Experiments were performed in triplicates and repeated to confirm the results.

### 2.14. GMSC Differentiation on Scaffolds

**Figure 1:**
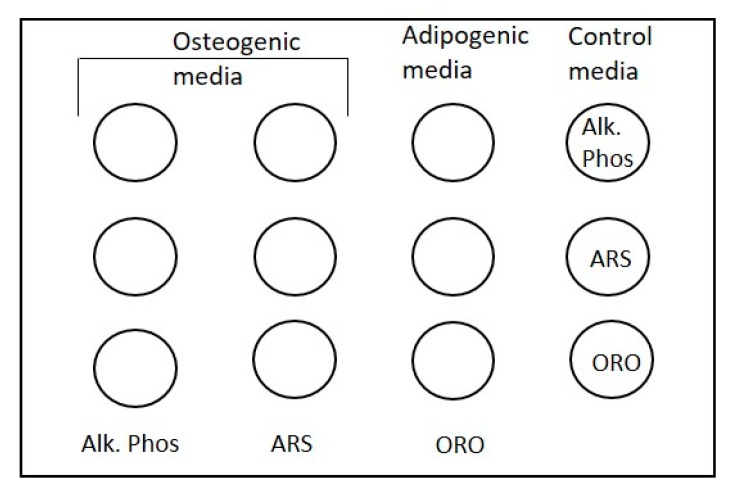
The setup for differentiation assay for GMSCs on electrospun scaffolds on a 12-well plate. Each well had 20,000 GMSCs overlaid on top of identical e-PCL scaffolds. Cells in the first two columns were incubated in osteogenic media; third column had adipogenic media and the fourth column had cells in maintenance (control) media. After three weeks, the cell-scaffold constructs were stained for Alk. Phos (alkaline phosphatase); ARS (alizarin red S) and ORO (Oil Red O). Control wells were also stained as specified. This setup allows for controls of media and stain specificity.

A total of 20,000 cells were placed in the center of the scaffolds in 100 μL of culture media for 24 h to facilitate cell attachment to scaffolds. At 24 h, the media was replaced with specialized osteogenic or adipogenic media and replaced every 3 days for a total of 3 weeks. Control wells had cells-scaffolds incubate in maintenance media. Osteogenicity was evaluated with staining by Alizarin Red and Alkaline Phosphatase, while Oil Red O verified adipogenicity. The hGMSCs and dGMSCs cultured in maintenance media served as controls. These were also stained for Alizarin red, Oil Red O, and alkaline phosphastase. The layout for the differentiation assay was modified from published protocol [21] and is given in Figure 1.

### 2.15. Statistical Analyses

Values were presented as means and standard deviation, where appropriate. Analyses that compared healthy and diseased tissue with respect to CFU efficiency, alkaline phosphatase production used analysis of variance, and significant differences were described using Tukey’s honest significant difference (HSD). All analyses were performed using SAS software (JMP version 10; SAS Institute, Inc., Cary, NC, USA). Cell proliferation assay on scaffolds were tested by a paired *t*-test for significant difference. Significance set a-priori at 0.05.

## 3. Results

### 3.1. Adherent Cells Isolated from Healthy and Diseased Gingiva Showed Characteristics of MSC

The Mesenchymal and Tissue Stem Cell Committee of the International Society for Cellular Therapy proposed these criteria [22] when defining human MSCs:

- Must be plastic-adherent under standard culture conditions
- Must express CD105, CD73, and CD90. MSCs should not express CD45, CD34, CD14, or CD11b, CD79alpha, or HLA-DR surface molecules and,
- Must differentiate into multiple lineages in vitro

#### 3.1.1. Adherent Cells from Both Diseased and Healthy Gingiva Exhibit CFU Activity, Although to Different Degree

**Figure 2:**
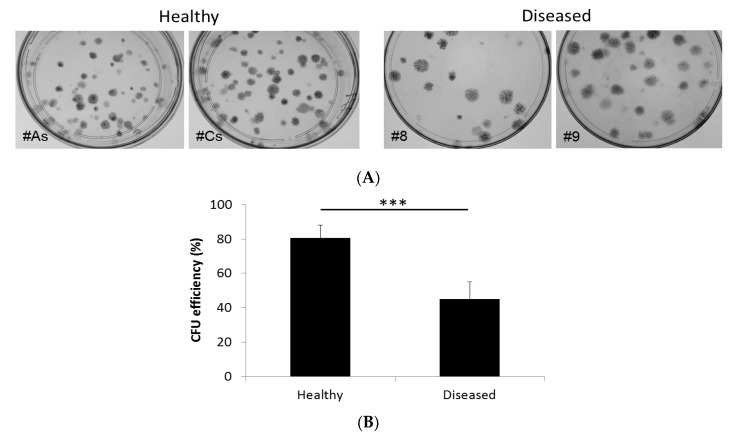
CFU efficiency adherent cells derived from healthy and diseased gingiva after 14 days of culture. (**A**) Representative photographs of the plates after staining with crystal violet; (**B**) Quantification of CFU efficiency shows a significant difference between the two types. *** *t*-test, two-tailed *p*-value <0.0001.

Cultures from both healthy and diseased gingival tissue formed adherent colony-forming units on plastic after 14 days incubation (Figure 2A). Qualitatively, the CFUs were less defined and fewer in diseased tissue compared to healthy gingiva. Counting of the individual colonies showed a significant difference in the CFU forming ability between the two types of tissues (Figure 2B). While cells from healthy gingiva patients exhibited an average CFU efficiency of 80.5%, cells from diseased gingiva had a CFU efficiency of 45% (*t*-test two-tailed *p*-value: <0.0001).

#### 3.1.2. Flow Cytometry: Adherent Cells from Both Tissues Express Cell Surface Markers for Adult MSC

**Figure 3:**
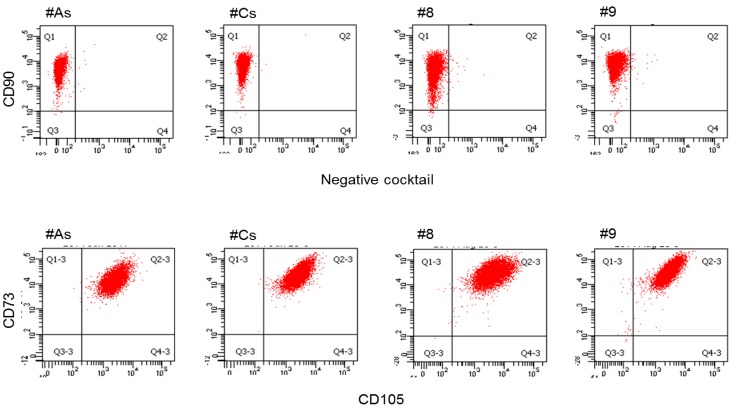
Flow cytometry data of cells derived from healthy (**left panel**) and diseased (**right panel**) gingiva both show identical expression of cell-surface markers characteristic of MSC. Cells were labeled with fluorescent conjugated antibodies and characterized using flow-cytometry.

Both populations of GMSCs analyzed were positive for the cell surface markers CD90 (Figure 3. top panel, *y*-axis), CD73 (Figure 3. bottom panel, *y*-axis), and CD105 (Figure 3. bottom panel, *x*-axis). Healthy and diseased GMSCs were negative for CD11b, CD19, CD34, CD45, HLA-DR (negative cocktail) (Figure 3. top panel, *x*-axis). Flow cytometric analysis showed no apparent phenotypic differences between healthy and diseased groups.

#### 3.1.3. Adherent Cells from Healthy Gingiva Showed Higher Osteogenicity, while Cells from Diseased Gingiva Showed Increased Adipogenesis

**Figure 4:**
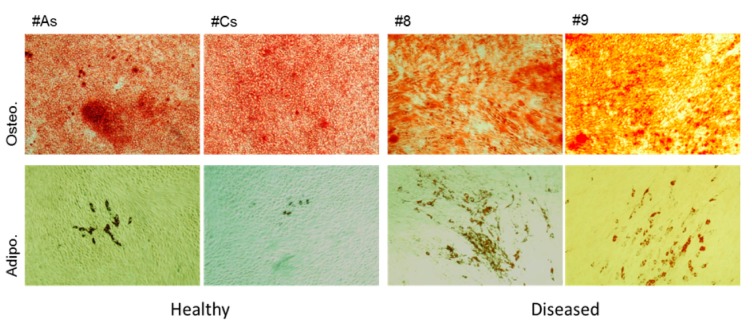
Multi-lineage differentiation of cells derived from healthy (**left**) and diseased (**right**) gingiva over 21 days. The top panel represents cells cultured in osteogenic media and stained with Alizarin red; the middle panel represents MSC cultured in adipogenic media, stained with Oil Red O, while the lower panel represents culture in maintenance media. All photographs were taken at the same magnification to illustrate sparse adipocytic differentiation compared to osteogenic differentiation.

Both populations of adherent cells underwent osteogenesis (Figure 4. Top panel); however, the cells derived from healthy gingiva had very high levels of mineralization, with a score of 5 (positive staining of entire well surface with virtually all cells producing mineralized nodules) while cells from diseased gingiva showed less apparent mineralization, with an average score of 4 (a high number of cells producing mineralized nodules).

While cells derived from healthy gingiva exhibited very few adipocytes in a few fields (score 1), cells derived from diseased gingiva showed a higher propensity for adipogenesis (score 4) with well-defined lipid droplets (Figure 4. middle panel).

Based on these results, we can safely conclude that the adherent cells derived from healthy and diseased gingiva can be recognized as MSCs. Henceforth, we will call these cell populations as gingiva-derived MSCs (GMSCs), specifically hGMSCs and dGMSCs to refer to healthy or diseased status of the donor site, respectively. It is important to realize that the cell populations differ in their propensity towards osteo- and adipo-genesis, at least on tissue culture plastic.

### 3.2. Alkaline Phosphatase Is Produced in Higher Levels in Healthy GMSCs (hGMSCs) Compared to Diseased GMSCs (dGMSCs)

**Figure 5:**
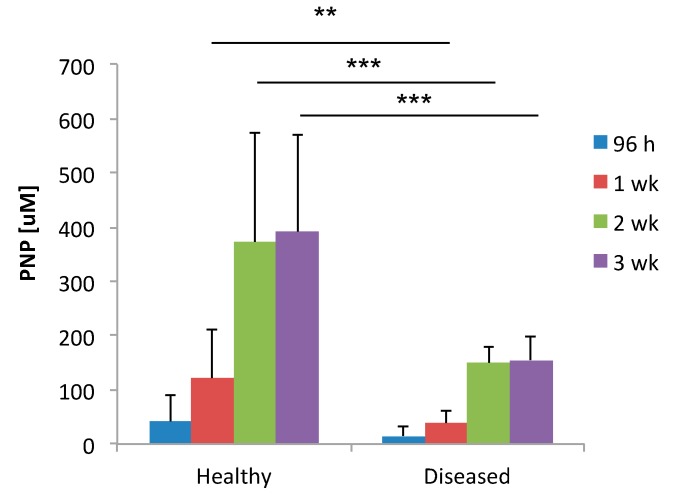
Alkaline phosphatase production in hGMSCs and dGMSCs. Cell number, assessed by MTS assay, was used to normalize the colorimetric signal prior to analysis. Assays were done at 72 h, 1 week, 2 weeks, or 3 weeks. In both groups, levels of ALP increased over time. There was significantly less ALP in the dGMSCs compared to that of the hGMSCs at 1 week, 2 weeks, and 3 weeks. (*t*-test, two-tailed *p*-values: ** <0.01, *** <0.001).

Levels of ALP, a marker of osteoblastic differentiation, were assessed by colorimetric assay (Figure 5). In both healthy and diseased groups, levels of ALP increased over time, appearing to level out by 3 weeks. There was significantly less ALP in diseased cells compared with that of healthy cells at 1, 2, and 3 weeks.

### 3.3. Electrospun Scaffold Characterization by SEM

**Figure 6:**
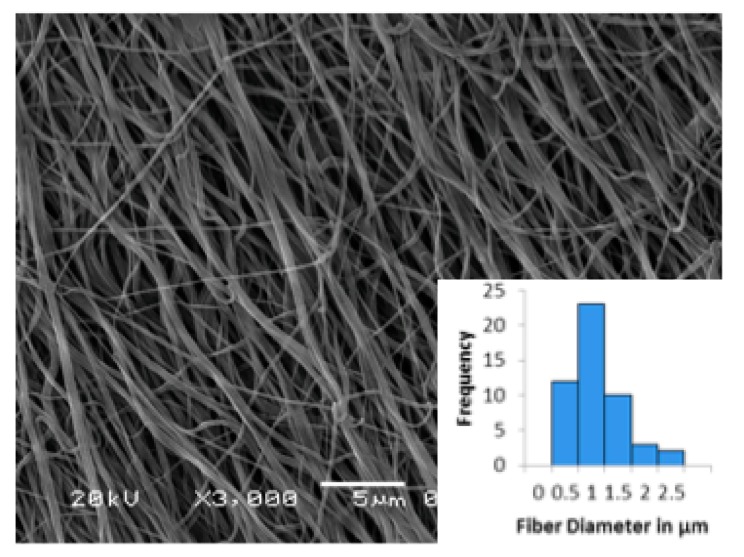
SEM of e-PCL fibers showing porous, fibrous morphology. Inset is the histogram of the fiber diameters of 50 randomly chosen fibers, showing the distribution. The average fiber diameter was 0.82 μm.

Porous, nanofibrous scaffolds were generated after the optimization of electrospinning conditions (voltage: 25 kV; Flow rate: 2 mL/h; Air gap distance: 12.5 cm). SEM analyses (Figure 6) revealed a broad distribution of fiber diameters ranging from 136 to 2200 nm with an average diameter of 0.82 ± 0.52 μm.

### 3.4. 24-Hours Cell Survival and 7-Days Proliferation on Electrospun Scaffolds

**Figure 7:**
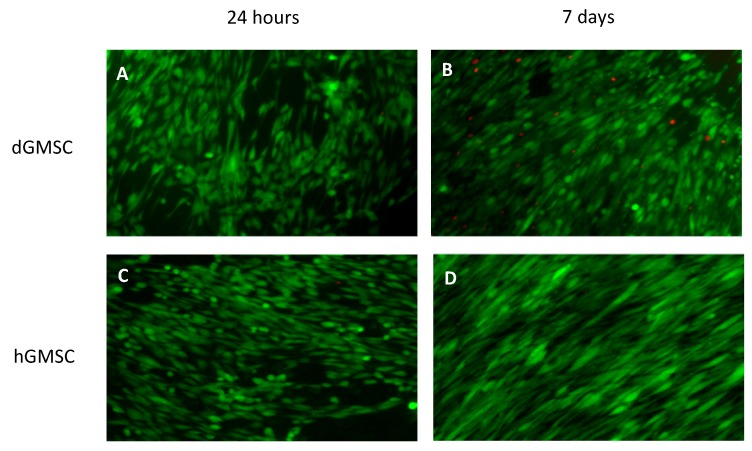
Live/Dead Assay of SEM of GMSC derived from diseased (dGMSC) and healthy (hGMSC) human gingiva at 24 h of culture. (**A**,**C**) represent the images at 24 h while (**B**,**D**) are taken at 7 days of culture.

**Figure 8:**
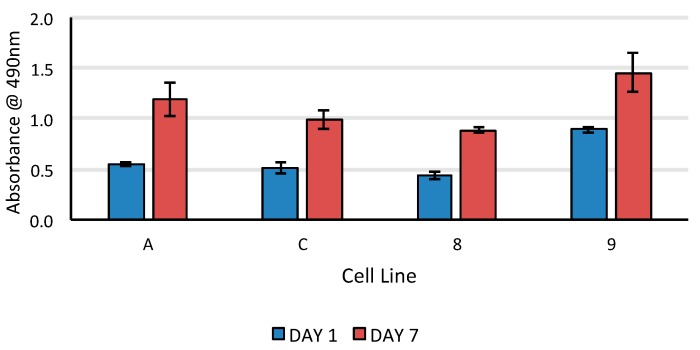
Cell proliferation as assessed by MTS in GMSC cultured on electrospun PCL scaffolds in 24 h and 7 days. There is clear evidence of cell proliferation over time irrespective of the health of donor gingival tissue even though there are differences in the degree of proliferation rates.

The GMSC from both the healthy and diseased gingiva showed a strong initial attachment and survival at 24 h as well as at 7 days evidenced by the Live/Dead Assay (Figure 7). This was consistent across different primary cell lines. We observed that the cells tended to align along the direction of the fiber, as has been confirmed in previous studies. When analyzing the cell proliferation over 7 days, we were able to see a significant increase in cell numbers compared to that at baseline (initial cell seeding density) irrespective of the cell line or the health status of the gingival tissue (Figure 8). There were, however, differences between individual cell populations due to intrinsic differences in primary cell lines. We did not compare the cell proliferation across different cell populations for the same reason.

### 3.5. GMSC Differentiation on Electrospun PCL Scaffolds

**Figure 9:**
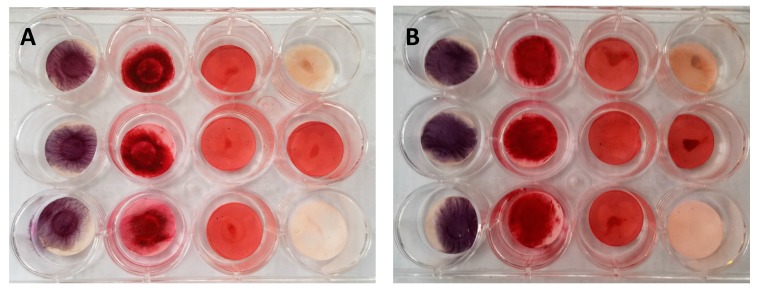
Results of 21-day differentiation assay of GMSCs; **Panel A**—dGMSCs and **Panel B**—hGMSCs. The setup outlined in Figure 1 was followed in these experiments. The first two columns represent cells cultured in osteogenic media while the third was cultured in adipogenic media and the fourth column represents control. Columns 1, 2s and 3 were stained for alkaline phosphatase, Alizarin red, and Oil Red O, respectively. The top well in the control was stained for alkaline phosphatase, middle well for Oil Red O, and bottom well for Alizarin red. As can be seen, **panel B** stains more strongly for osteogenic markers compared to **panel A**, suggesting increased osteogenic potential for hGMSCs. Oil Red O demonstrates non-specific binding of the stain to the cell-scaffold construct.

The behavior of the GMSC on electrospun scaffolds was interesting. Both the hGMSCs and dGMSCs showed a strong tendency for osteogenesis as reflected by the high intensity staining for Alkaline Phosphatase and Alizarin Red (Figure 9). The setup of the experiment allowed us to rule out non-specific staining of cell-laden scaffolds. Control wells (GMSCs cultured in maintenance media) free of staining indicate that electrospun PCL scaffolds supported osteogenic differentiation. This was true of both h- and d-GMSCs.

When the cells were cultured on adipogenic media, the results were not very clear. This was because of non-specific binding of Oil Red O to the cell-scaffold constructs in the control wells.

## 4. Discussion

Bone grafting is the standard of care for treating larger than critical-sized defects, common after infections, surgery, or trauma. Options include autografts and allografts; the former is associated with additional surgery and increased morbidity while the latter is associated with risk of disease transmission. Given these limitations associated with bone substitutes, tissue engineering has emerged as an alternative approach. Tissue engineering draws on principles of cell biology, developmental biology, and biomaterials to fabricate new structures to replace lost or damaged tissues [23]. The goal of bone engineering is to restore the structure and function of the missing tissue using a combination of scaffolds, cells, and signaling molecules. Upon implantation, these cell-scaffold constructs are expected to induce host cells to lay down new bone and eventually turn over and integrate with the host bone [7].

MSCs are attractive in scaffold-based tissue engineering because of their unique properties: clonal expansion and multi-lineage differentiation. These properties allow for harvesting of relatively small numbers of MSCs, expanding them in the lab, and directing differentiation by exposing them to specific biochemical signals [24,25,26]. MSC-derived cells can be seeded in biocompatible scaffolds, which can be shaped into the anatomical structure and then surgically implanted to heal the defect [27].

Even though bone marrow and adipose tissue have been the preferred sources for MSCs, because of ease of harvest and potential for autologous use, they have several disadvantages. First, bone marrow-derived stem cells BMSCs are associated with extremely low yields (0.001% to 0.01%) that require large quantities of bone marrow aspiration with attendant donor site morbidity [28]; both BMSCs and adipose-derived stem cells ADSCs are present as mixed cell populations and require several passages in culture to purify and enrich the MSCs [29,30]. Another limitation of BMSC is the functional heterogeneity amongst seemingly pure preparations as determined by clonal expansion assays [31].

Gingiva refers to specific oral tissue that forms a soft tissue cuff around the tooth and forms a first line of defense against physical, chemical, and biological assault. It is a part of the oral mucosal immunity. The intrinsic wound healing properties of gingiva—reduced inflammation, rapid re-epithelialization, and fetal-like scarless healing—are driven primarily by the mesenchymal stem cells that reside in the lamina propria of the gingiva.

GMSCs lend themselves to rapid in vitro expansion owing to shorter population doubling and relative homogeneous cell population. It has been shown that adherent cells isolated from a small piece of gingival tissue (~2 × 2 mm^2^) usually reached confluence (~1–2 × 10^6^ cells) after culture for 10~14 days. GMSCs were shown to be similar to BMSCs in terms of CFU efficiency (4–6%), population doublings, and ability to maintain early phenotypes at passage six. GMSCs were found to be superior to BMSCs in terms of proliferation rate (40 h vs. 80 h), maintenance of normal karyotype and telomerase activity in long-term cultures, and not being tumorigenic [32,33].

Within our study, we found differences in both of these aspects between GMSCs derived from healthy and diseased gingiva: hGMSCs had higher CFU and more osteogenic tendency compared to those of dGMSCs (which demonstrated more adipogenicity). It is interesting to explore the reasons for these observed differences. While this could be attributed to primary cell lines derived from innately different donors, role of disease status should merit consideration. This is because of increased understanding of the role of epigenetics in the development and progression of periodontal disease [34,35,36]. Recently, Xu et al. reported that the MSC fate was also affected by epigenetic factors [37]. In a comparative study of MSCs derived from bone marrow and adipose tissue from the same patient, they found hypomethylation of Runx2 promoter (an osteogenesis specific transcription factor) in BMSCs as well as of the PPARγ promoter (an adipogenesis specific transcription factor) in ADSC. Since the degree of methylation is inversely related to gene expression, these epigenetic differences accounted for differential behavior of these cell lines. We hypothesize that differences in behavior between GMSCs derived from diseased and healthy gingiva could be attributed to epigenetic changes associated with chronic periodontitis. If DNA methylation studies verify these changes, it could permit resetting of the epigenetic memory of GMSCs derived from diseased gingiva towards osteoblastic lineage resulting in favorable bone regeneration. Since periodontitis can be localized to one to few teeth, it will be interesting to compare the behavior of GMSCs harvested from healthy and diseased sites within the same donor.

In this study, we demonstrate that GMSCs can be isolated from healthy and periodontally diseased tissues. MSCs from each of these sources share key characteristics such as fibroblast-like appearance, adherence to plastic, expression of certain cell surface antigens (CD73, CD90, CD105), and lack of expression of others (CD11b, CD14, CD19, CD34, CD45, CD78) [9]. Our results indicated no difference in the expression of cell surface markers between diseased and healthy cells, as confirmed by prior studies [13,38]. Whilst the above criteria fit well for cells isolated from gingival tissues, it is important to realize that satisfying the above mentioned requirements cannot reliably confirm MSCs from other cell types such as fibroblasts [15]. The true test of MSCs is in fact their multi-lineage differentiation potential under appropriate conditions, whereas fibroblasts do not differentiate into osteoblasts and adipocytes [39].

The potential of mesenchymal stem cells in therapeutic interventions is already being explored in a variety of clinical scenarios that have limited treatment options. Preclinical and clinical data support the use of these cells in plastic surgery, orthopedics, myocardial infarction, graft vs. host disease, and autoimmune diseases [40]. It is important to realize that most of the clinical data comes from BMSCs or ADSCs. However, with the potential of MSCs beginning to be understood, it is important to explore alternate sources of adult MSCs. In this sense, GMSCs represents a readily available cell source that can be potentially used in alveolar bone regeneration, the most challenging situation in chronic periodontitis. Recent studies showing GMSCs can be successful in generating functional constructs with alginate microspheres [41,42,43]. By demonstrating the viability of 3D tissue constructs using GMSCs and e-PCL scaffolds, as well as their ability to support osteogenesis, our study expands the application of this novel cell source.

## 5. Strengths, Limitations, and Future Research Directions

- Generalizability of results: One of the strengths of the study is that the GMSCs evaluated are primary cell sources that are not modified (transformed) in any way. Using finite lines for MSCs is challenging because they are slow and hard to establish, impose limits on the passage numbers that can be used for differentiation, and are expensive to maintain (with specialized media). However, these are the biologically more appropriate cells to study because of their relevance in clinical translation. The results of this study should be interpreted with caution because the data is derived from four primary cell sources that have inherent variability. Future efforts could involve establishing a gingival tissue repository and its distribution to laboratories for evaluating the behavior of these cells. As mentioned above, another option would be to study MSCs derived from healthy and diseased gingiva from the same patient.
- Data quality: The current study used qualitative measures (staining for Alizarin red, Oil Red O, Alkaline phosphatase) to study GMSC differentiation. We adopted this approach because staining can provide early proof-of-concept information about the behavior of GSMCs on electrospun scaffolds. Careful experimental setup with proper controls allowed us to visually verify the differentiation of GMSCs. However, lack of quantitative measures is a limitation and future studies with robust quantifiable data will improve the strength of conclusions. More detailed experiments involving RT-PCR and Western blotting can provide insights into the mechanisms controlled the fate of MSCs.
- A minor limitation of this study could be not validating the GMSCs for their chondrogenic lineage. Detailed characterization of GMSCs by previous research groups has established that GMSCs do possess multi-lineage potential and, hence, these tests were not repeated in our study. Since our primary goal was to evaluate effectiveness of GMSCs in bone engineering, we felt that doing chondrogenic assays would not add significantly to the scientific merit of the study.

## 6. Conclusions

Bone tissue engineering involves the triad of scaffolds, cells, and signaling molecules. While electrospun PCL has remained a popular choice in bone engineering, gingiva has been recently identified as a tissue source for MSCs. This study extends the scope of GMSCs in bone engineering by investigating the potential of MSCs derived from healthy and diseased gingival tissues on electrospun matrices. Results from 2D culture clearly indicate superior osteogenicity of hGMSCs while the 3D culture on PCL scaffolds showed comparable osteogenicity in dGMSCs and hGMSCs. Future studies should explore the role of electrospun matrices in directing differentiation, compared to tissue culture plastic substrate, particularly in the context of cell signaling. Overall, the study results indicate that diseased gingiva could serve as a viable source of GMSCs given the fact that these tissues are routinely discarded during surgery. Evaluation of osteogenicity of GMSC-laden electrospun scaffolds in appropriate animal models can provide critical insights into their potential for bone regeneration. Such information would be important prior to clinical application in humans.
